# Isolated systematized nevus Unius Lateris: a case report

**DOI:** 10.1093/omcr/omae046

**Published:** 2024-05-20

**Authors:** Rima Alhalabi, Youlla Oun, Kinda ALshawa

**Affiliations:** Department of Dermatology and Venereology, Damascus University, Damascus, Syria; Department of Dermatology and Venereology, Damascus University, Damascus, Syria; Department of Dermatology and Venereology, Damascus University, Damascus, Syria

## Abstract

Nevus unius lateris is a rare congenital cutaneous hamartoma derived from the ectoderm and is considered as a verrucous variant of the epidermal nevus. Although it can affect any body part, it rarely involves the head and neck region. When the nevus becomes widely distributed, it usually associated with systemic involvement known as epidermal nevus syndrome. We report here a case of a 7-year-old male patient with a diagnosis of systematized nevus unius lateris, with bilateral involvement of the head and neck and without associated comorbidities, owing to its rarity.

## INTRODUCTION

Verrucous epidermal nevus (VEN) is a common benign, noninflammatory malformation that presents at birth or develops in early childhood. However, its diffused linear distribution known as systematized nevus unius lateris is rare. There have been approximately 200 cases reported in the medical literature [1]. They present as linear or whorled, skin colored to gray-brown and black verrucous papules, which may coalesce to form plaques that tend to follow the lines of Blaschko and are usually associated with systemic abnormalities [1, 2]. We present the case of a 7-year-old male patient diagnosed with an isolated systematized nevus unius lateris. To the best of the authors’ knowledge, this is the first report of such a case in the Syrian dermatologic literature.

## CASE REPORT

**Figure 1:**
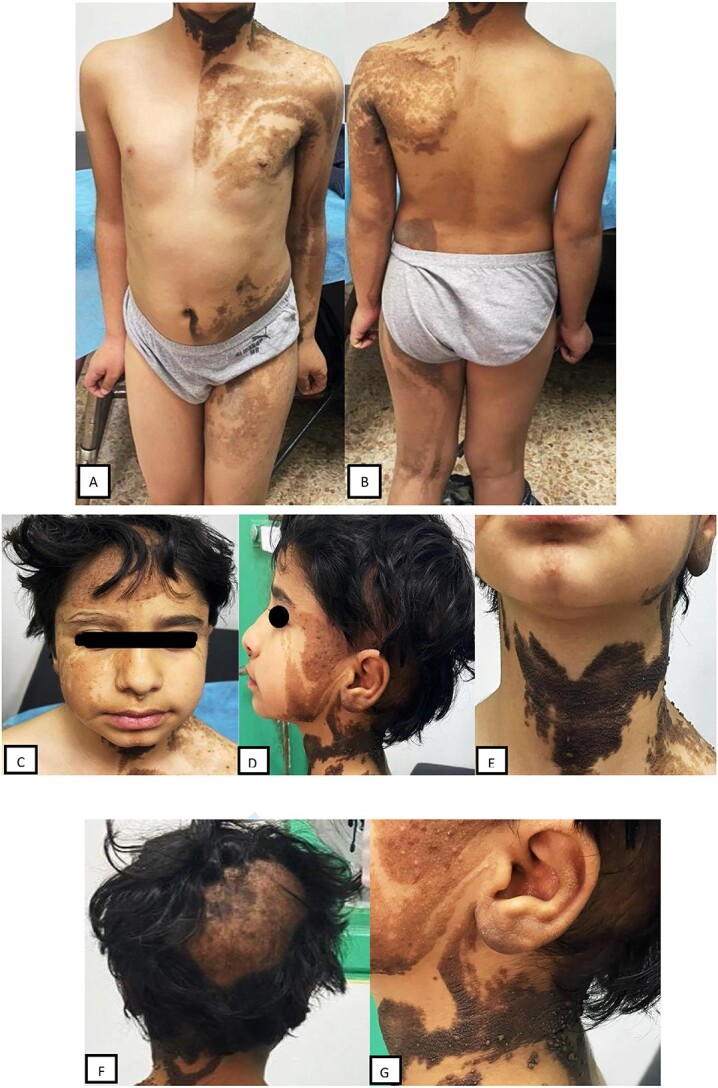
Hyperpigmented verrucous plaques on (**A**) left side of anterior trunk, left upper and lower limbs; (**B**) left side of posterior trunk, left upper and lower limbs. (**C,D**) bilateral lesions on face. (**E**) bilateral lesions on neck. (**F,G**)Sparsely distributed hairs over some parts of the scalp.

A 7-year-old male patient presented to the Dermatology Outpatient Department with asymptomatic hyperpigmented verrucous plaques predominantly covering the left half of the trunk, left upper and lower limbs, but sparing palms and soles (Fig. 1).

Lesions were also present bilaterally on the face, neck and scalp (Fig. 1).

The described lesions were present at birth and progressively increased in size and thickness with age.

The growth and developmental history were normal and there was no history of seizures, learning or hearing difficulties, altered vision or skeletal abnormalities. There was no family history of similar lesions.

Examination showed multiple pigmented, hyperkeratotic papules and plaques arranged in streaks and whorls over the face, neck and left side of the trunk and extremities. The oral and genital mucosa were found to be normal.

On hair examination, the hairs were thin, hypopigmented, sparse and short over some parts of the scalp, while the rest of the scalp had well-developed terminal hairs (Fig. 1). No systemic abnormality was detected.

**Figure 2:**
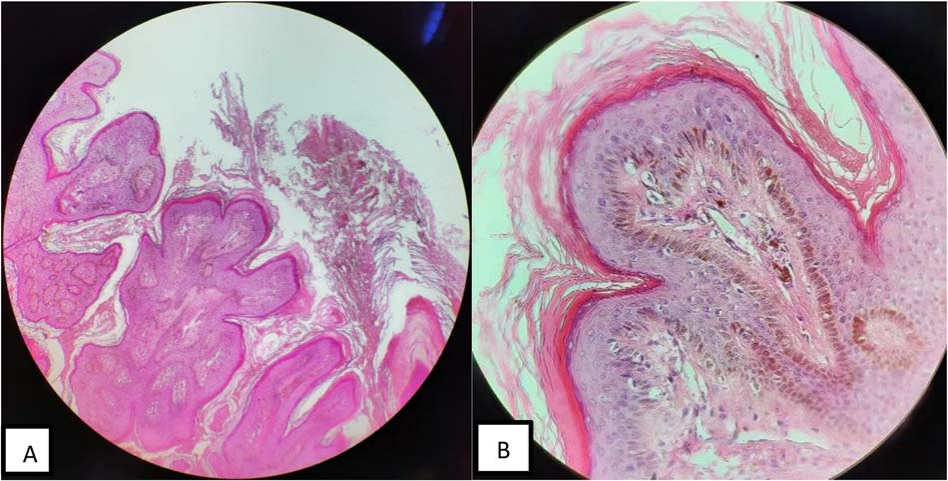
Histologic findings (H&E, ×100). (**A**) Presence of hyperkeratosis, irregular acanthosis and papillomatosis. (**B**) high basal melanin content.

Routine investigations, including auditory and ophthalmological examinations, brain MRI and skeletal survey were normal. Histopathological examination of lesional biopsy showed hyperkeratosis, acanthosis, papillomatosis and an increase in basal melanin (Fig. 2).

Based on clinical and histopathological features, a diagnosis of isolated systematized nevus unius lateris was made. The patient did not receive any treatment but was advised to see a doctor when changes ocuur in the skin nevus.

## DISCUSSION

Verrucous epidermal nevus (VEN) is a rare cutaneous hamartoma derived from the embryonic ectoderm, with onset usually appearing at birth but may become more obvious later in life [2, 3]. VEN is characterized by skin-coloured to tan verrucous papules and plaques that usually arise on the trunk and limbs and are uncommon over the head and neck region. In rare cases, VEN may affect the face, scalp and lobes [1, 4].

VEN can be categorized based on its clinical appearance, either as localized linear leasions that occur along the lines of Blaschko or as systematized leasions when there are more than one linear lesion.

The systematized lesions are further divided into two uncommon forms: ‘Nevus Unius Lateris’ which affects one-half of the body, and ‘Icthyosis Hystrix’ which shows bilateral distribution [4, 5]. The prevalence of VEN is approximately 1:1000 [1, 5], but the specific variant of nevus unius lateris represents only 0.01% of all VEN cases [1].

Most cases with extensive distribution are generally associated with systemic involvement, such as neural, ocular and skeletal abnormalities, such cases are called epidermal nevus syndrome or Solomon syndrome [2]. Less commonly, this entity can manifest as an isolated finding [1]. Neurological disorders are the most common, which can include mental retardation, seizure, epilepsy and brain tumors. Ophthalmological abnormalities may present as coloboma, epidulbar choristomas and corneal opacities. Kyphoscoliosis, vit D-resistant rickets, growth retardation and auditory disturbances have been described in several cases [5].

Clinical examination is the gold standard for diagnosis to date, although a skin biopsy may be performed to confirm the diagnosis, rule out other causes of verrucous dermatosis following the lines of blaschko and for research purposes [1, 6]. Histological findings may include hyperkeratosis, papillomatosis, acanthosis, increased melanin in the basal layer and rarely epidermolytic hyperkeratosis [7].

Our patient was evaluated as an isolated extended nevus unius lateris that presented with typical lesions widely distributed on one half of the body (trunk, extremities, neck, face, scalp and lobe of the ear), following the lines of Blaschko. No systemic abnormalities were observed by the examination. Biopsy of the lesions also showed typical findings, as an indicated above, which confirmed the diagnosis.

The main clinical differential diagnosis is the verrucous stage of incontinentia pigmenti, which is an x-linked dominant multisystemic genodermatosis. it presents in the first weeks of life, and Skin lesions progress through four different stages (vesicular, verrucous, hyperpigmented and hypopigmented/atrophic), following the lines of Blaschko. Eosinophilic spongiosis is shown on histology [3]. Skin lesions in our patient did not precede any vesicular stage and he did not have any systemic involvement.

There is no ideal therapy for VEN, and the treatment is mainly symptomatic for cosmetic purposes [7]. Several therapies have been reported with variable results, including surgical and non-surgical excision such as topical and oral retinoids, topical corticosteroids, 5-fluorouracil, calcipotriol, podophyllin [3], cryotherapy [9] and carbon dioxide laser [8], which are considered less aggressive methods.

Because there is a possibility of malignant transformation in epidermal nevus, we ask the patient to consult a dermatologist periodically when any changes occur in the cutaneous lesions [10].

## CONCLUSION

Verrucous epidermal nevus, as an isolated systematized nevus unius lateris, has rarely been described. It is important for clinicians to diagnose these cases because, although it is rarely an isolated finding, most patients with extensive unilateral skin involvement are associated with neural, ocular, skeletal and auditory abnormalities. Therefore, auditory and visual screening tests, computed tomography and skeletal survey should be done at birth and later in life to rule out any associated disorders.
